# Focal Fibrocartilaginous Dysplasia: Site-Specific Patterns of Presentation and Management—A Narrative Review

**DOI:** 10.3390/jcm15135200

**Published:** 2026-07-03

**Authors:** Viola Sbampato, Ahmer Ahmad Khan, Elio Paris, Andreas Tsoupras, Wassim Ben Abdennebi, Ardit Ademi, Oscar Vazquez, Giacomo De Marco, Dimitri Ceroni

**Affiliations:** 1 Paediatric Orthopaedics and Traumatology Unit, University Hospital of Geneva, CH-1205 Geneva, Switzerlandoscar.vazquez@hug.ch (O.V.);; 2 Faculty of Medicine, University of Geneva, CH-1211 Geneva, Switzerland

**Keywords:** paediatric orthopaedics, focal fibrocartilaginous dysplasia, toddler, tibia, genu varum/valgum, guided growth, narrative review

## Abstract

**Background/Objectives**: Focal fibrocartilaginous dysplasia (FFCD) is a rare, benign developmental disorder of the growing skeleton first described 40 years ago. It is characterised by a fibrocartilaginous tether adjacent to the physis, which disrupts symmetrical growth and leads to progressive angular deformity. The aim of this review was to define site-specific clinical patterns and management principles for FFCD to optimise patient outcomes. **Methods**: We conducted a narrative review of more than four decades of published literature on FFCD. All identified English-language case reports, case series, and review articles were analysed to synthesise evidence on clinical presentation, anatomical location, natural history and treatment strategies. **Results**: To date, 169 cases have been reported, with approximately two-thirds involving the proximal tibia. Tibial lesions typically present in toddlers as unilateral genu varum and, in most cases, demonstrate spontaneous remodelling and complete resolution. In contrast, femoral and upper-limb lesions rarely resolve spontaneously and often progress, thereby warranting earlier and more invasive management. Radiographic findings are highly characteristic, most commonly showing a cortically based metaphyseal lucency with a sclerotic rim. These features are generally considered sufficient for diagnosis, usually eliminating the need for biopsy. Management has evolved towards a tailored approach, consisting of observation for tibial lesions with potential for spontaneous resolution and timely surgical intervention for femoral or upper-limb lesions at risk of progression or joint compromise. **Conclusions**: Despite the advances made in recent decades, FFCD remains a distinctive yet heterogeneous condition in paediatric orthopaedics. This narrative review summarises more than four decades of published literature, including case reports, case series, and review articles, with particular attention to site-specific clinical patterns and their implications for optimising management.

## 1. Introduction

Focal fibrocartilaginous dysplasia (FFCD) is a rare, benign developmental disorder of the growing skeleton that primarily affects infants and young children. First described in 1985 by Bell et al. in the proximal tibia, it is characterised by a fibrocartilaginous lesion adjacent to the growth plate, which disrupts symmetrical physeal growth and results in progressive angular deformities [1]. Although often described as a “tumour-like” lesion due to its radiological appearance, FFCD is not a true neoplasm but rather a localised growth disturbance [2].

The proximal tibia is by far the most commonly involved site, accounting for over 60% of all reported FFCD cases to date [3]. Less frequently, FFCD affects the distal tibia, distal femur, ulna, proximal humerus, radius, phalanges, and, very rarely, the vertebrae or ribs. Despite this wide anatomical distribution, the underlying pathophysiological mechanism appears consistent: a localised fibrocartilaginous “tether” that interferes with symmetrical bone growth [4].

Given the rarity of FFCD and the evolving understanding of its natural history, several aspects remain central to optimising diagnosis and guiding appropriate therapeutic management. These include epidemiological evidence such as age of onset and anatomical predilections; etiopathogenic hypotheses; clinical presentation and site-specific deformity progression; and pathognomonic radiological and histological features.

This narrative review offers a site-by-site analysis of FFCD, aiming to clarify site-specific clinical patterns and diagnostic criteria. It emphasises the role of conservative management when appropriate and defines the circumstances in which surgical intervention becomes justified.

## 2. Materials and Methods

This narrative review is based on a comprehensive literature search conducted in PubMed, Embase, and Cochrane Library databases. The search covered the period from January 1985 to December 2025, and the last database search was performed on 31 December 2025. Search terms included combinations of “focal fibrocartilaginous dysplasia”, “fibrocartilaginous dysplasia”, “fibrous periosteal inclusion”, “tibia vara”, “angular deformity”, “genu varum”, “paediatric bone deformity”, and “growth modulation”. This search strategy was supplemented by a manual search of the reference lists of retrieved articles to identify additional relevant studies.

Studies eligible for inclusion were original clinical investigations addressing the epidemiological, pathophysiological, diagnostic, or therapeutic aspects of FFCD, including prospective or retrospective cohort studies, case series, and isolated case reports. No restrictions were imposed regarding patient age, anatomical site of involvement, or clinical presentation. Conference presentations, abstract-only proceedings, instructional lectures, reviews, letters to the editor, animal studies, and in vitro experimental research were excluded. Publications lacking an English abstract or an accessible translation were also excluded. Historical non-English publications, including articles published in French, Spanish, and Italian, were identified through database searches and manual reference screening. These studies were included whenever sufficient clinical information could be extracted.

Study selection was performed in two stages, consisting of title and abstract screening followed by full-text assessment. Each article was reviewed independently by two reviewers (VS and GDM) according to the predefined eligibility criteria. Disagreements regarding study eligibility were resolved through discussion and consensus. When consensus could not be reached, a third reviewer (DC) performed an independent reassessment and made the final decision. A total of 102 records were identified and screened, of which 62 articles were ultimately included in the review. Findings were synthesised qualitatively to identify site-specific patterns relevant to diagnosis and management.

Ethical approval was not required for this narrative review in accordance with local legislation and institutional requirements. Written informed consent for the publication of potentially identifiable clinical images originating from our institution and included in this article was obtained from the participants’ legal guardians. 

During manuscript preparation, ChatGPT (version 5) was used exclusively to improve the readability and language of the text. Its use was limited to editorial support and did not contribute to the literature search, study selection, evidence synthesis, interpretation of the findings, or formulation of the scientific conclusions. All AI-assisted revisions were critically reviewed and incorporated at the authors’ discretion, and the authors take full responsibility for the final manuscript.

## 3. Results of Literature Research

**Figure 1:**
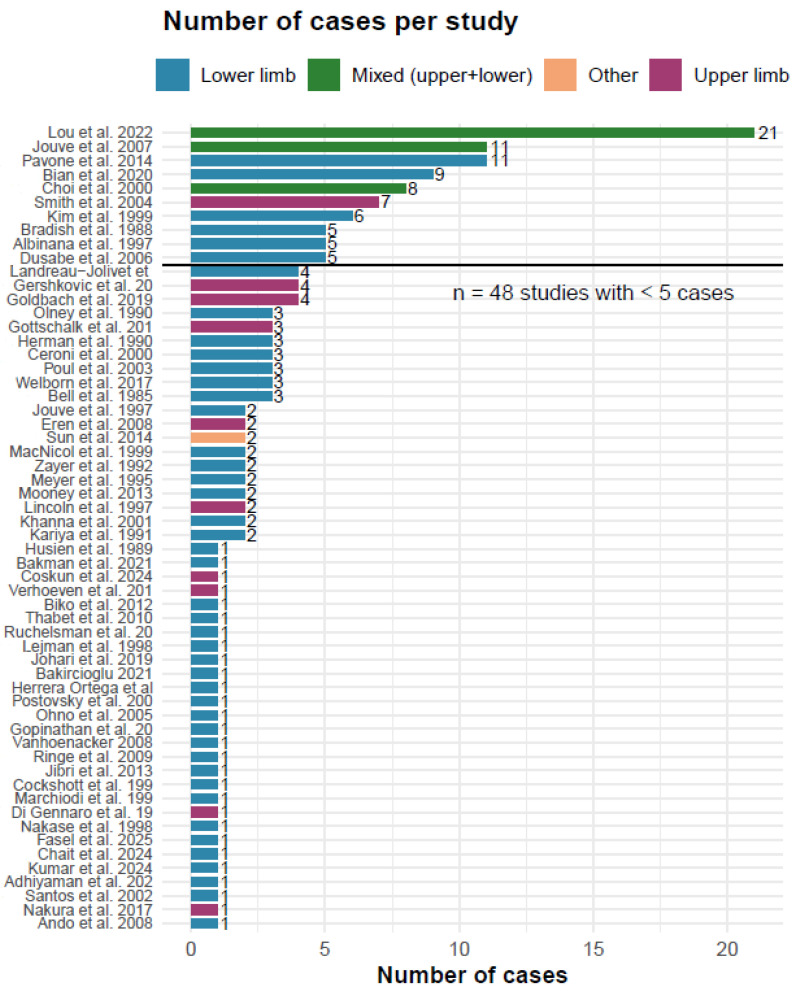
Graphical representation of the number of cases reported in each included study [2,3,4,5,6,7,8,9,10,11,12,13,14,15,16,17,18,19,20,21,22,23,24,25,26,27,28,29,30,31,32,33,34,35,36,37,38,39,40,41,42,43,44,45,46,47,48,49,50,51,52,53,54,55,56,57,58].

Our search strategy identified 102 records, all of which underwent title, abstract, and full-text screening. Of these, 62 articles met the eligibility criteria and were included in the narrative review, with 58 reporting individual FFCD cases. The available literature was composed predominantly of isolated case reports and small case series: 48.3% of included publications were single case reports, 48 studies reported fewer than five cases, and only three studies described more than ten patients. The distribution of reported cases across the included studies is illustrated in Figure 1. In addition, nearly 80% of publications focused primarily on treatment, whereas the remaining studies were mainly descriptive. Most reports provided follow-up exceeding two years, particularly in cases managed conservatively or surgically.

## 4. Historical Review

FFCD was first described in a case series published in 1985 by Bell et al., reporting three children with unilateral tibia vara associated with a distinctive fibrocartilaginous cortical defect in the proximal medial tibia [1]. The authors hypothesised that this abnormal growth resulted from a failure of mesenchymal precursor differentiation, thereby defining the entity and giving it the name that remains in use [1,5]. In the late 1980s and early 1990s, additional reports confirmed the predominance of tibial involvement and noted that in some children the deformity spontaneously corrected with growth [6,7,8]. These early reports emphasised the characteristic radiological appearance of FFCD, which permitted differentiation from Blount’s disease—a condition with more diffuse metaphyseal changes and physeal involvement—as well as from other conditions including physiological bowing, non-ossifying fibroma, fibrous cortical defect, fibrous dysplasia, and osteofibrous dysplasia [5,9,10]. By the mid-1990s, femoral involvement had also been documented [11], and subsequent publications extended the spectrum to the upper limb, including the humerus and ulna [9]. Ulnar disease, first described in 2005, was recognised as a distinct presentation often associated with forearm shortening and secondary radial head dislocation [4,12,13,14,59]. Together, these reports broadened the anatomical distribution of FFCD and established the framework for its clinical variability. In parallel, advances in imaging, particularly MRI, facilitated diagnosis without biopsy [15,60]. Management also shifted over time: initially broad indications for surgical excision gave way to more conservative strategies, with the introduction of new techniques such as guided growth and piezosurgical excision reported in select cases [10,16,17,18,19,61].

## 5. Epidemiology

**Figure 2:**
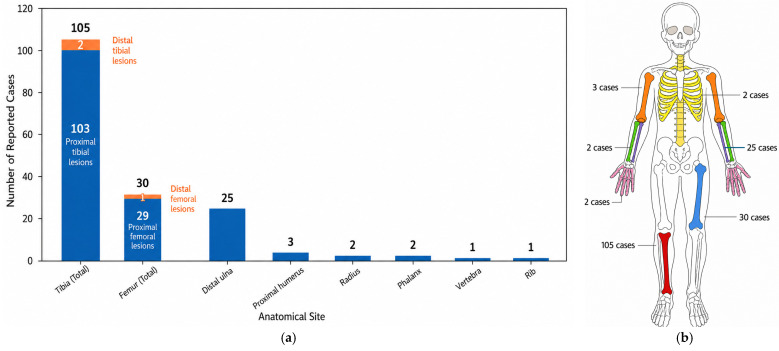
(**a**) Anatomical distribution of FFCD cases across reported skeletal sites. Bars are arranged in descending order according to the number of reported cases, with exact case numbers (n) displayed above each bar. (**b**) Schematic summary of the anatomical distribution of FFCD across the skeleton. Colours indicate the affected anatomical sites and their corresponding number of reported cases: tibia (red, n = 105), femur (blue, n = 30), ulna (green, n = 25), humerus (orange, n = 3), radius (purple, n = 2), phalanges (pink, n = 2), vertebra (yellow, n = 1), and rib (yellow, n = 1).

To date, FFCD has been documented in only 169 cases in the literature. Approximately two-thirds of these (105 cases; 62.1%) involve the tibia. Of the tibial cases, only two have been reported in the distal tibia [15,20], four on the lateral side of the proximal metaphysis [3,20,21,22], while the vast majority—99 cases (96%)—arise from the medial side of the proximal tibial metaphysis, typically resulting in characteristic varus deformities [1,2,3,5,6,7,8,9,10,15,16,17,20,21,22,23,24,25,26,27,28,29,30,31,32,33,34,35,36,37,38,39,40,41,42,58]. Beyond the tibia, FFCD has also been reported in other lower-limb sites, most notably the proximal and distal femur, with 30 cases described to date [2,4,5,10,15,19,21,42,43,44,45,46,47,48,49,50,60,61]. Upper-limb localisations—affecting the proximal humerus, distal ulna and radius, or proximal phalanges—remain exceptional, with approximately 34 cases reported [4,5,10,12,13,14,15,18,51,52,53,54,57]. Rarely, FFCD has also been described in the axial skeleton, with isolated cases involving the vertebrae and ribs [52]. The anatomical distribution of FFCD cases across reported skeletal sites is illustrated in Figure 2a, with a schematic summary provided in Figure 2b.

The age at diagnosis may be highly variable. The condition has been reported as early as birth and as late as 38 years of age, although the vast majority of patients are infants and toddlers [10,60]. In the lower limb, most patients are diagnosed before the age of 4, with the mean age for tibial FFCD reported at 21.4 months [3,17,35]. By contrast, upper-limb cases are more frequently described in older children and adolescents; proximal humeral lesions have been reported in patients between 11 and 17 years of age [10,18].

With respect to sex distribution, early reports suggested a slight male predominance [3]. However, findings have been inconsistent across published series, with some studies reporting an approximately equal male-to-female ratio and others observing varying degrees of male predominance [17,48]. Overall, the available evidence does not support a consistent sex distribution pattern. Racial and ethnic data have not been systematically reported in the literature, precluding any conclusions regarding potential racial or ethnic predisposition.

## 6. Etiopathogenesis

The precise origin of FFCD remains debated, and multiple theories have been proposed. In the lower limb, one of the earliest explanations proposed that tibial FFCD resulted from an abnormal differentiation of the mesenchymal precursor tissue at the insertion of the pes anserinus, leading to local overproduction of fibrocartilage and interference with symmetrical physeal growth [1]. A later and widely accepted hypothesis describes FFCD as a “fibrous periosteal inclusion,” in which abnormal anchorage of the periosteum to the metaphyseal cortex prevents its normal sliding during bone growth, effectively acting as a partial epiphysiodesis whereby the uninvolved side of the physis continues to grow normally while the tethered side lags, producing angular deformity [5]. Alternative theories have emphasised direct growth plate involvement [15]. Some authors suggest that physeal cartilage remnants or displaced cartilage islets incorporated into the metaphyseal cortex may disturb endochondral ossification, creating asymmetric growth centres [20]. Others propose that localised necrosis of the physis, triggered by prenatal or perinatal trauma, infection, or mechanical overload, may heal with fibrocartilaginous tissue that persists as a tether [1,5,29,30,35,42,62].

Among the proposed mechanisms, the “fibrous periosteal inclusion” hypothesis is currently the most widely accepted, as it provides a plausible explanation for both the characteristic anatomical location of the lesion and its growth-restricting effect on the physis. Nevertheless, no single theory has been conclusively proven, and the precise etiopathogenesis of FFCD remains uncertain. Current understanding is derived largely from histopathological observations and isolated clinical reports, reflecting the rarity of the condition and the absence of dedicated molecular studies.

In the upper limb, the etiopathogenesis is thought to be broadly similar to that of the lower limb, although certain site-specific features have been described. In the ulna, FFCD typically arises at tendon insertions or fibrous anchorage points, where dense fibrous or fibrocartilaginous tissue extends across the physis and restricts longitudinal growth [13].

## 7. Clinical Presentation and Natural History

The natural history of FFCD is difficult to define within a uniform timeline, as data from published reports are heterogeneous and sometimes contradictory [3,10]. Nevertheless, clear site-specific patterns emerge, reflecting the interplay between the tethering fibrocartilaginous lesion and the growth potential of the affected physis. A summary of the clinical presentation and natural history according to anatomical location is provided in Table 1.

The lower limb is the most frequently affected site, with lateral metaphyseal lesions of the proximal tibia giving rise to genu valgum and medial metaphyseal lesions to genu varum, also known as the classic presentation of FFCD [5]. The deformity typically becomes apparent during the first year of ambulation and worsens in approximately 40% of cases during the initial 8–9 months of weight-bearing [1,28]. Clinically, in these early years, parents often notice unilateral bowing of the leg and a mild limb-length discrepancy [4].

Nevertheless, the prognosis in these cases remains favourable, with spontaneous correction typically beginning around 24 months of age and continuing through 3–4 years of age [5,6]. Spontaneous correction occurs in roughly 45% of patients, with median recovery times of about 57 months [10,17]. The knee angulation therefore gradually resolves without intervention, supported by the intrinsic remodelling capacity of the proximal tibial physis.

However, not all tibial lesions follow a benign course: some remain static, while others progress, with an increasing metaphyseal–diaphyseal Levine-Drennan angle (L–D angle) serving as a warning sign. An angle exceeding 20 degrees or persistent worsening despite observation indicates an unfavourable evolution [5]. Failure to improve within 6–12 months is likewise considered concerning. In such cases, surgical release or detethering may accelerate correction and prevent progression to severe deformity [5,17]. By contrast, distal femoral and tibial FFCD rarely remodel spontaneously, and the resulting knee or ankle angular deformities almost always require surgical management [5,10,19,60].

Lesions of the upper limb are rarer and also follow a much less favourable course [13]. Clinically, their presentation is often delayed, as the resulting limb-length discrepancy and angular deformities cause less functional impairment than those affecting the lower limb [10,13,35,53]. For this reason, children tend to present later, after parents notice mild deformities or asymmetries in the absence of prior trauma [13].

The ulna is the most frequently affected upper-limb bone, with onset typically occurring between 2 and 6 years of age [4,13,14,54]. The deformity often progresses steadily during growth, almost invariably leading to radial bowing with secondary radial head subluxation or dislocation, with restriction of pronation–supination and pain or discomfort [12,13,14].

Unlike tibial lesions, spontaneous resolution of ulnar FFCD is exceptional, and the majority of reported cases have required surgical treatment [14]. Other upper-limb sites, though rarer, also show unfavourable behaviour [54]. Proximal humeral lesions carry a particular risk of growth arrest, with untreated cases showing severe shortening—sometimes of several centimetres at maturity—along with angular deformity and impaired shoulder function [53]. Radial lesions present in infancy or early childhood as distal forearm angulation, sometimes mistaken for fracture malunion [54]. Phalangeal involvement, though rare, is typically evident at birth as finger shortening, deviation, or angulation [54].

Axial localisations are extremely rare and, to date, have been described only in a single report. This case involved a thoracic vertebra and an adjacent rib in a young adult, where the clinical presentation was dominated by persistent back pain rather than angular deformity [52]. Given this solitary observation, the clinical features and natural course of axial FFCD remain poorly defined [52].

## 8. Radiological Investigations

FFCD has a highly characteristic imaging appearance that is considered pathognomonic, allowing diagnosis in most children without the need for biopsy. On plain radiographs, the lesion appears as a well-defined, cortically based radiolucent defect at the metaphysis adjacent to the physis, with a characteristic sclerotic rim outlining the concavity of the deformity [5,9,10,34,60]. The cortex is usually thinned but preserved, and there are no aggressive features such as soft-tissue mass, cortical destruction, or malignant-type periosteal reaction. While the majority of cases are radiographically straightforward, occasional reports describe periosteal thickening or a florid periosteal response, particularly in the tibia [5,9].

**Figure 3:**
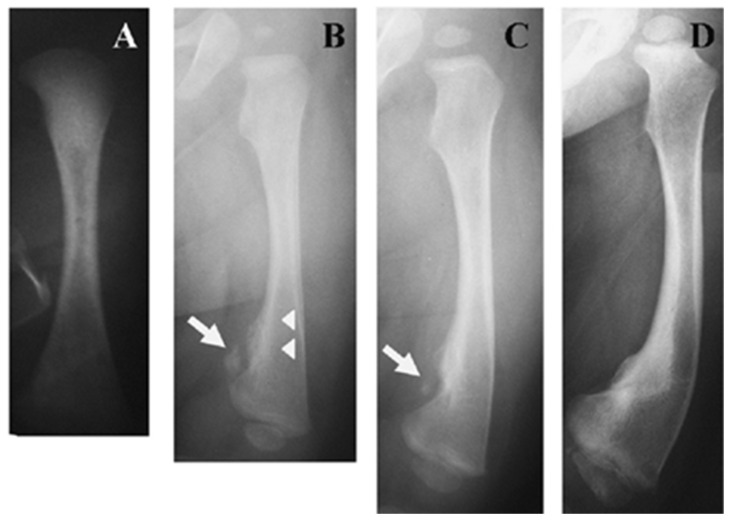
Radiographic progression of angular deformity in FFCD of the distal left femur. Reproduced from Tohoku J. Exp. Med. (Ando et al., 2008) [60] with permission from Tohoku University Medical Press. Anteroposterior radiograph of the left femur. (**A**) Two weeks after birth. Abnormal change was not apparent. (**B**) Ten weeks. A sclerotic change (arrowheads) with bony island (arrow) became apparent. (**C**) One year. The bony island became smaller (arrow) and varus deformity was apparent. (**D**) Two years. Varus deformity progressed up to 40 degrees.

The natural radiographic progression of angular deformity in FFCD is well illustrated in the distal left femur (Figure 3). Initially unremarkable two weeks after birth (Figure 3A), sclerotic changes with a bony island become apparent by ten weeks (Figure 3B). At one year, the bony island diminishes while varus deformity emerges (Figure 3C), progressing to 40 degrees by two years (Figure 3D).

**Figure 4:**
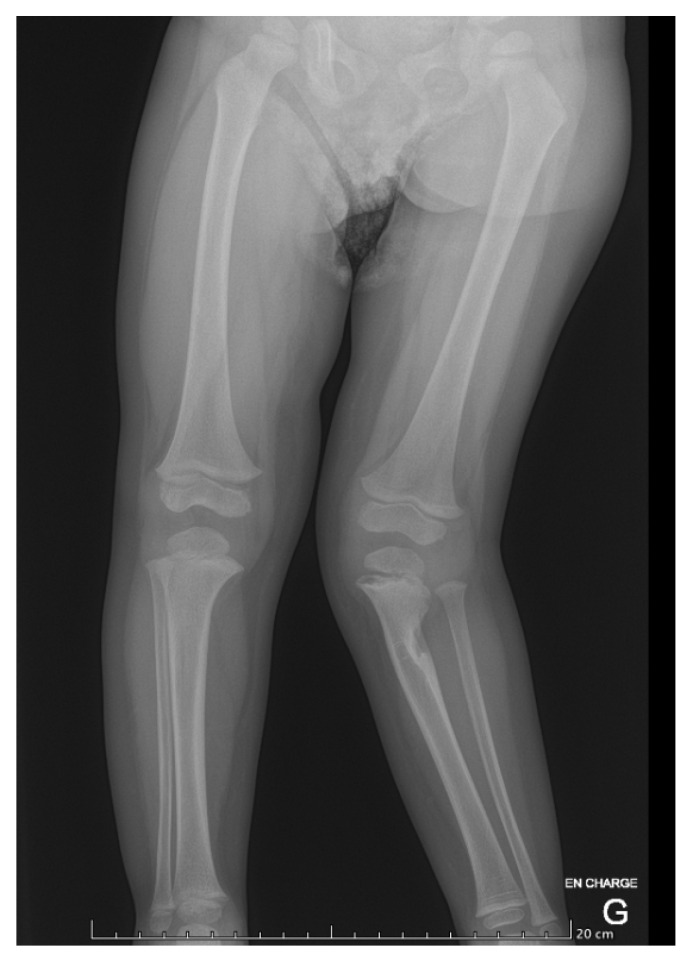
Radiographic imaging (anteroposterior weight-bearing view) demonstrating an unusual valgus deformity of the left proximal tibia in FFCD, characterised by a lateral radiolucent metaphyseal defect with surrounding cortical condensation and irregularity. The letter “G” on the radiograph indicates the left side (“gauche” in French).

Beyond the typical varus alignment, unusual valgus deformity of the left proximal tibia can also occur, characterised by a lateral radiolucent metaphyseal defect with surrounding cortical condensation and irregularity (Figure 4).

**Figure 5:**
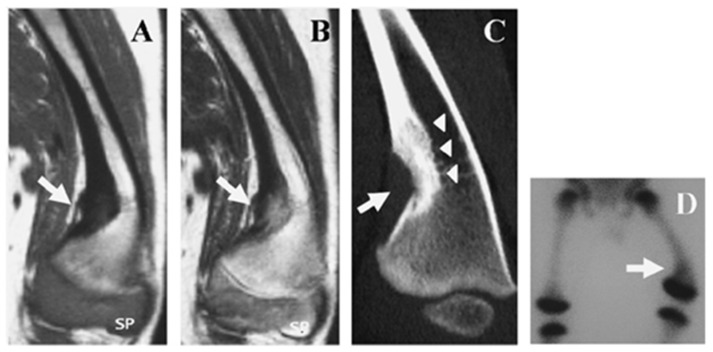
Imaging findings of FFCD in the distal left femur using different modalities. Reproduced from Ando et al., 2008 [60], with permission. Radiological findings of the lesion. (**A**,**B**) Magnetic resonance imaging showed lower signal intensities on T1-weighted images (arrow) and higher signal intensities on T2-weighted images (arrow) than muscles. An abnormal extraosseous mass was not apparent. (**C**) Computed tomography showed bony defect (arrow) with marginal sclerosis (arrowheads). (**D**) Bone scan with 99mTechnetium showed a mild uptake in the distal femur (arrow).

Recognizing this age–site–pattern triad is critical to avoid unnecessary invasive procedures and to support conservative management. Advanced imaging is seldom required but may be useful in atypical sites or when the diagnosis is uncertain. Early reports suggested that MRI provided limited additional diagnostic value in atypical cases. However, recent papers demonstrated that MRI was particularly useful for investigating the cortical location of the lesion and for assessing the fibrous or fibrocartilaginous tether [15,28]. Classically, the lesion demonstrates low signal on T1-and T2 weighted sequences in areas corresponding to the fibroligamentous tether at the cortical lucency, intermediate signal in adjacent sclerotic regions, and areas of higher signal corresponding to cartilage [12,15,28]. As shown in the distal left femur (Figure 5), MRI reveals lower signal intensities on T1-weighted images and higher signal intensities on T2-weighted images compared to muscle, without an abnormal extraosseous mass (Figure 5A,B).

**Figure 6:**
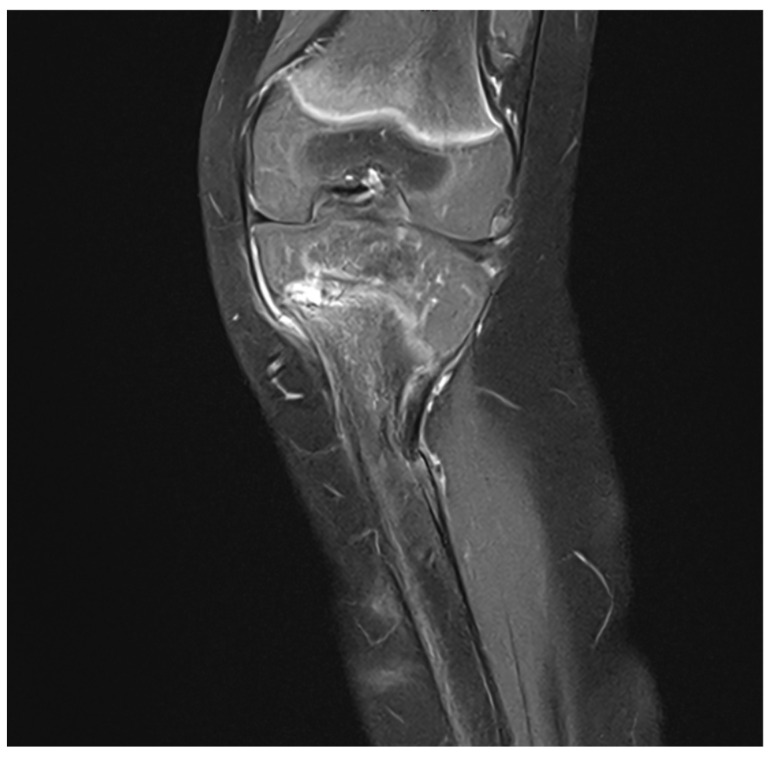
Coronal MRI (T2-weighted fat-suppressed sequence) demonstrating FFCD as a low-signal-intensity lesion in the lateral metaphysis of the left proximal tibia.

Coronal T2-weighted fat-suppressed MRI further demonstrates FFCD as a low-signal-intensity lesion in the lateral metaphysis of the left proximal tibia (Figure 6).

CT is generally not required, as the fibrocartilaginous tissue cannot be distinguished from adjacent muscles and tendons. However, it can be valuable in selected cases for delineating the extent of cortical involvement [5,15,54]—for example, showing a bony defect with marginal sclerosis in the distal femur (Figure 5C). Ultrasound has occasionally been used, showing a hypoechoic cortex resembling a gapping fracture [13,35]. Bone scintigraphy, when performed, typically shows only mild uptake, as demonstrated with 99mTc in the distal femur (Figure 5D).

## 9. Histopathology

FFCD demonstrates a characteristic but variable histological profile across the different anatomical sites. In the lower limb, lesions are composed of a mix of dense fibrous tissue, fibrocartilage, hyaline cartilage, and combinations of these [1,6,8,10,11,16,18,22,32,45,54]. Tibial lesions often contain less rigid and more heterogeneous tissue, which may explain their tendency to remodel spontaneously with growth [2]. In contrast, femoral lesions frequently reveal thick fibrous cords that traverse the physis and insert into the epiphysis, creating a fixed tether that prevents spontaneous correction [2,10,11,19,44,46,48,54]. Microscopically, FFCD lesions typically show immature fibrocartilage islands embedded in fibrous stroma, disruption of the normal sequence of endochondral ossification, and a paucity of osteoblastic activity, with no evidence of osteoclastic resorption, atypia, or malignant features [2]. Occasional areas of necrosis, dystrophic calcification, or ossification may also be observed, but the overall picture is that of a benign developmental disturbance rather than a neoplastic process [2].

Upper-limb lesions share many of these features but exhibit distinct patterns that correlate with their clinical course. Ulnar FFCD, the most frequent form in the forearm, often consists of a fibroligamentous or fibromuscular tether anchored to the metaphyseal cortex, sometimes extending across the physis to adjacent carpal bones [4,13,14]. Histological findings range from purely dense fibrous tissue to admixtures with fibrocartilage and hyaline cartilage [2]. In some cases, only fibrous periosteal-like tissue has been identified, indicating variability in tissue composition along a spectrum. The rigidity of this tether explains why ulnar deformities tend to progress, resulting in bowing, shortening, and secondary radiocapitellar joint incongruity [13]. Unlike tibial FFCD, spontaneous resolution in the upper limb is exceptional, and the histopathological architecture supports the concept of a persistent mechanical barrier to growth [2,10].

## 10. Management

As stipulated above, the management of FFCD depends on the site of involvement, severity of deformity, and natural course. Because the condition is benign and often self-limiting, treatment has shifted from aggressive correction towards more conservative and individualised approaches [3,5,10].

The indications for surgery remain somewhat heterogenous and seem closely linked to the site affected by the FFCD, with lesions involving the upper limb more frequently leading to surgical intervention. Surgery may also be offered to accelerate the healing process or to obtain histological confirmation in atypical cases. Most of the time, it is primarily the lack of resolution or even the progression of deformity, its severity, and its functional repercussions that constitute the main indications for surgery. In some cases, surgery may also be offered when the deformity is deemed functionally unacceptable at the insistent request of the parents. However, neither a pathological fracture nor pain alone appears, to our knowledge, to be a recognised indication for surgical treatment.

Tibial lesions are the best studied and illustrate the range of options. In many children, clinical and radiographic follow-up for up to 24 months is sufficient, particularly when the L–D angle is less than 30 degrees and there is no functional limitation [33]. However, recommendations vary among authors. While some authors support observation for angles below 30 degrees [33], others advocate a shorter observation period of 6–12 months and consider an L–D angle exceeding 20 degrees to be a predictor of unfavourable progression [5,10]. Bracing, splinting and shoe elevation have been attempted in some cases but have not been shown to modify the natural history of FFCD [32]. When deformity worsens or fails to improve, authors have described curettage of the fibrocartilaginous focus as an effective and relatively simple intervention to consider. Such surgery is particularly indicated when the child is above the age of 24 months and has an L–D angle greater than 20 degrees that has widened over the preceding 6–12 months [5,10]. Removal of the tether allows the physis to resume symmetrical growth, often leading to rapid correction within a year [5].

Compared with osteotomy, curettage is less invasive, achieves correction rates of up to 2 degrees per month, and avoids complications such as peroneal nerve palsy, recurrence, or overcorrection from the Cozen phenomenon, namely a late-onset post-traumatic valgus deformity associated with a proximal tibial fracture [5,6,24,42]. Hemi-epiphysiodesis and guided growth have also been proposed, but they do not address the tether, may fail to correct the true source of rotation, and are technically challenging in children under 4 years of age [20,23,24]. Osteotomy remains an option in severe or neglected cases where deformity has become established, but it is generally reserved as a secondary procedure [3,10,19,60].

As discussed previously, femoral lesions behave differently. Since spontaneous improvement is exceptional and most cases progress without intervention, compromising joint mechanics and alignment, early surgery is recommended [5,10,19,48,60]. Curettage and excision of the fibrous tether, usually combined with corrective osteotomy, can restore alignment and prevent worsening deformity. If corrective surgery is delayed, a more complex reconstruction may be necessary [5,10,19,48,60].

Upper-limb involvement presents unique challenges and generally requires more active intervention, as spontaneous resolution is reported far less frequently [51,55]. The ulna is the most common site and the most clinically significant: progressive bowing and shortening predispose to radial head dislocation, making early surgery advisable [13,59,63]. Excision of the tether, sometimes combined with osteotomy or gradual ulnar lengthening, maximises remodelling potential and preserves radial head stability [13]. Once dislocation has occurred, only salvage procedures remain, such as one-bone forearm creation, radial head resection, or bony synostosis, all of which compromise rotation [13,18,19]. Nakura’s review found that 10 of 14 patients (71%) with ulnar FFCD required surgical treatment; among these surgically managed patients, 9 of 10 had developed radial head subluxation or dislocation before intervention [12]. Alternative techniques such as isolated ulnar lengthening across the growth plate show promise in correcting deformity without osteotomy-related complications, although long-term data are limited [13,18,19]. Exceptional spontaneous resolution of ulnar lesions has also been reported [10,18,54].

Radial lesions are rare and may be observed if mild, though corrective osteotomy is an option for progressive deformity [54]. Humeral cases are uncommon and usually stable, but despite reports of successful conservative management, many patients develop significant shortening, with osteotomy and fixation reserved for symptomatic deformities [18,53]. Phalangeal FFCD, typically congenital, is notoriously resistant to surgery, with frequent recurrence despite osteotomy [54].

Axial cases involving the vertebrae or ribs are exceedingly rare, and although biopsy may be required to exclude more aggressive pathology, subsequent curettage and observation have been reported, although no standardized approach exists to date [52].

## 11. Conclusions

Focal fibrocartilaginous dysplasia is a rare developmental disorder characterised by a fibrocartilaginous tether that disrupts bone growth and produces localised deformity. Diagnosis is usually established based on characteristic radiographic features, making biopsy unnecessary. Its natural history is strongly site-dependent: tibial lesions in young children frequently remodel spontaneously and are best managed with careful observation, whereas femoral and upper-limb lesions rarely resolve and tend to progress. In these locations, early surgical release of the tether—often combined with corrective procedures—is generally indicated to prevent irreversible deformity and joint dysfunction. Recognition of these site-specific patterns underscores the importance of including FFCD in the differential diagnosis of atypical paediatric bone deformities. A clear understanding of FFCD is essential to avoid misdiagnosis, guide appropriate management, minimise unnecessary surgical intervention, and prevent long-term morbidity in children. However, the available evidence remains limited by the predominance of isolated case reports and small case series, with substantial heterogeneity in reporting and follow-up. Consequently, the conclusions drawn from the current literature should be interpreted with appropriate caution.

## Figures and Tables

**Table 1:** Summary of clinical presentation and natural history of FFCD according to anatomical location.

**Anatomical Site**	**Typical Age at Presentation**	**Clinical Presentation**	**Natural History**	**Management**
Proximal tibia (medial metaphysis)	Infancy to early toddler age	Unilateral genu varum, mild limb-length discrepancy, onset during early ambulation	Frequently favourable; spontaneous remodelling in ~45% of cases, typically beginning around 24 months of age [5,6,10]	Initial observation is generally favoured; surgical detethering may be considered if deformity progresses, metaphyseal–diaphyseal angle > 20°, or no improvement after 6–12 months [5,6]
Proximal tibia (lateral metaphysis)	Infancy to early toddler age	Genu valgum (rare presentation)	Limited data; remodelling possible but less predictable [20,22]	Close monitoring is advised; surgery may be considered if progression occurs
Distal tibia	Childhood (rare)	Ankle varus or valgus deformity	Poor spontaneous correction reported [20]	Surgical intervention is frequently reported because spontaneous correction appears uncommon
Femur	Early childhood to adolescence	Progressive knee or limb malalignment	Unfavourable; spontaneous resolution exceptional [10,19]	Early surgical excision of the tether has been advocated by several authors, often combined with corrective osteotomy
Ulna	Early childhood	Progressive forearm bowing, shortening, limited rotation; risk of radial head dislocation	Progressive in most cases; spontaneous resolution rare [14]	Early surgical intervention should be considered to prevent progressive forearm deformity and radiocapitellar instability.
Humerus	Late childhood to adolescence	Limb shortening, angular deformity, often mild symptoms	Variable; growth arrest and shortening common [18]	Observation possible; surgery for symptomatic or progressive deformity
Radius	Infancy to childhood	Local angular deformity, shortening	Generally unfavourable [54]	Surgical correction if functional impairment
Phalanges	Typically evident at birth	Shortening, deviation, or angulation	Generally unfavourable [54]	Surgical correction if functional impairment
Axial skeleton	Adolescence to adulthood (exceptional)	Pain rather than deformity	Poorly defined [52]	Individualized management: biopsy often required to exclude aggressive pathology

## Data Availability

No new data were created or analyzed in this study. Data sharing is not applicable.

## Abbreviations

The following abbreviations are used in this manuscript:

FFCD	Focal fibrocartilaginous dysplasia
L–D angle	Levine–Drennan metaphyseal–diaphyseal angle
MRI	Magnetic Resonance Imaging
CT	Computed Tomography
T1	T1-weighted magnetic resonance imaging sequence
T2	T2-weighted magnetic resonance imaging sequence
GNAS	Guanine nucleotide-binding protein alpha subunit
